# Sinomenine Ameliorates Colitis-Associated Cancer by Modulating Lipid Metabolism via Enhancing CPT1A Expression

**DOI:** 10.3390/metabo12100946

**Published:** 2022-10-05

**Authors:** Jing Zhang, Dan Huang, Yue Dai, Yu-Feng Xia

**Affiliations:** 1 Department of Pharmacognosy, School of Traditional Chinese Pharmacy, China Pharmaceutical University, Nanjing 211198, China; 2 Department of Pharmacology of Chinese Materia Medica, School of Traditional Chinese Pharmacy, China Pharmaceutical University, Nanjing 211198, China

**Keywords:** sinomenine, colitis-associated cancer, lipid metabolism, UPLC-QTOF-MS/MS, cell proliferation

## Abstract

Colitis-associated cancer (CAC), arising from long-lasting intestinal inflammation, is a common type of colorectal cancer. Sinomenine (SIN), the major active compound of *Sinomenium acutum*, displays excellent antitumor activity. In modern pharmacological research, SIN has been proved to arrest proliferation of human colon cancer cells in vitro, but its functional role and specific mechanism in CAC were still elusive. This study explored the molecular mechanism of SIN on CAC. The results showed that orally administered SIN could decrease the occurrence and development of CAC. Metabolomics results revealed SIN could reprogram metabolism in CAC mice by reversing 34 endogenous metabolites. Importantly, the most prominent metabolic alteration was lipid metabolism. Mechanistically, SIN improved lipid metabolism by enhancing the expression of CPT1A in CAC mice. Moreover, the inhibitory effect of SIN on the proliferation of human colon cancer cells was blunted via CPT1A inhibitor. The results of this study added further evidence of the molecular mechanisms that allow SIN to exert anti-CAC effect by facilitating lipid metabolism and reaffirmed its potential and distinctive role as a chemopreventive agent in CAC.

## 1. Introduction

Colorectal cancer (CRC) is the third most common and deadly cancer globally [1]. Colitisassociated cancer (CAC), as a common and aggressive form of CRC, is triggered by persistent intestinal inflammation. In recent years, the incidence rate and mortality rate of CRC have been increasing around the world [2]. In addition to genetics and environment, unfavorable and established risk factors, such as obesity, lack of physical exercise, excessive alcohol intake and smoking, directly affect intestinal regeneration and tumorigenesis [3,4]. Currently, surgery combined with chemotherapy and radiotherapy in clinic serve as the predominant method for CRC treatment [5,6]. Despite progress in surgical technology and therapy options, the mortality rate of CRC remains high in patients with advanced disease, and their prognosis is still worse, causing adverse reactions, such as liver metastasis, drug resistance, nausea, vomiting, weight loss and anemia [7,8]. On this account, the new preventive strategy is essential for the treatment of CAC.

**Figure 1:**
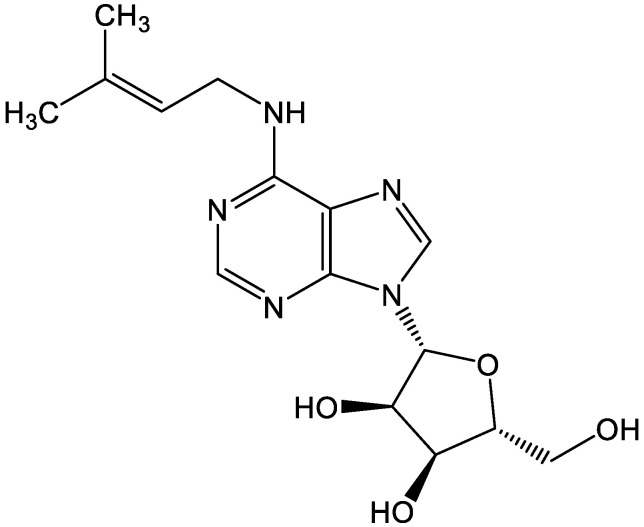
The chemical structure of sinomenine.

Recently, traditional Chinese medicine has garnered increasing interest for its definite curative effect, few side effects and low economic costs. Sinomenine (SIN, Figure 1), an active ingredient derived from *Sinomenium acutum*, possess multi-target anticancer activity [9]. Distinct mechanisms have been proposed for the inhibition of SIN on tumor formation, such as direct cytotoxicity, restriction of proliferation, and induction of autophagy [10,11,12,13,14,15]. These findings highlight that it holds the capacity to be developed as a potent cancer killer. Despite the fact that emerging studies have described SIN dampening the growth of colon carcinoma cells, whether it exerts an anti-CAC role in vivo and its mechanism remain to be elucidated [16,17].

Metabolomics, focusing on monitoring endogenous metabolites, could reflect the impact of disease course and drug target on the human body. Mounting evidence indicated that metabolomics has been extensively exploited as an available tool to investigate CRC, liver cancer, bladder cancer, etc. [18,19,20]. Although multiple altered metabolites in lipid, bile-acid, amino-acid, and other significant metabolite perturbations in various biological biofluids and tissues of CRC patients have been proposed, their exact mechanism is still unclear [21,22]. Here, the protective effect of SIN against CAC was evaluated, and an untargeted metabolomics approach based on ultra-performance liquid chromatography coupled with quadrupole time-of-flight mass spectrometry (UPLC-QTOF-MS/MS) was utilized to investigate the underlying mechanism. Subsequently, molecular biology technology was applied to analyze the alteration of the related molecular pathway in vivo. Finally, 3-(4,5-dimethylthiazol-2-yl)-2,5-diphenyl tetrazolium bromide (MTT) assay was performed to further excavate the deep mechanisms of SIN.

## 2. Material and Methods

### 2.1. Chemicals and Reagents

SIN (purity > 98%) was supplied by Nanjing Dilger Medical Technology Co., Ltd. (Nanjing, China). Azoxymethane (AOM), etomoxir (ETX) and dimethyl sulfoxide were produced by Sigma-Aldrich (Saint Louis, MO, USA). Dextran sulfate sodium (DSS) (molecular weight: 36–50 kDa) was the product of MP Biomedical (Irvine, CA, USA). HiScript^TM^Q RT SuperMix and Hieff^TM^ qPCR SYBR^®^ Green Master Mix were developed by Abm Biotechnology Co., Ltd. (Shanghai, China). GAPDH (1:10,000 dilution) was produced by Sangon Biotech Co., Ltd. (Shanghai, China). Methanol and acetonitrile were obtained from Merck Drugs & Biotechnology (Darmstadt, German). Formic acid was the product of Aladdin Bio-Chem Technology Co., Ltd. (Shanghai, China). L-2-chloro-phenylalanine was supplied by Shanghai Yuanye Biotechnology Co., Ltd. (Shanghai, China). TRIzol reagent was produced by Invitrogen (Carlsbad, CA, USA). NP-40 buffer was supplied by Nanjing SunShine Biotechnology Co., Ltd. (Nanjing, China). The BCA protein assay kit was developed by Nanjing Beyotime Biotechnology Co., Ltd. (Nanjing, China). Antibodies against carnitine palmitoyltransferase 1A (CPT1A) (for WB, 1:1000 dilution) and lysophosphatidylcholine acyltransferase 3 (LPCAT3) (for WB, 1:1000 dilution) were supplied by Cloud-clone Technology Co., Ltd. (Wuhan, China). Enzyme-linked immunosorbent assay (ELISA) kits were obtained from Shanghai Tongwei Biotechnology Co., Ltd. (Shanghai, China).

### 2.2. Animals

Six-week-old male C57BL/6J mice, weighing 18–20 g, were obtained from the Comparative Medicine Center of Yangzhou University (Yangzhou, China). Mice were housed with environmental temperature at 22 ± 2 °C. The design and experiment of animal models were approved by the Ethics Committee of China Pharmaceutical University (Approval No: 2022-05-013).

### 2.3. Induction of CAC and Treatments

The AOM/DSS-induced mouse model is widely exploited to assess the occurrence, progression and chemopreventive agents in CAC. The procedure used for the induction of CAC model by AOM/DSS was as previously described [23]. All mice were randomly divided into the following three groups: WT group (*n* = 8), AOM/DSS group (*n* = 11) and AOM/DSS + SIN group (*n* = 10). On day 1, the mice in AOM/DSS group and AOM/DSS + SIN group were injected with AOM (10 mg/kg, i.p.). After 1 week, the AOM/DSS group and AOM/DSS + SIN group were allowed access to 2% DSS for 7 days. Then, they were fed with water for another 14 days. This process was cycled three times. The mice in the AOM/DSS + SIN group were orally administered with 120 mg/kg SIN orally once a day, and the mice in the WT and AOM/DSS group were given an equal volume of vehicle. All samples including serum and colon tissues were placed at −80 °C until analysis.

### 2.4. Colonic Histological Analysis

The distal sections of the mouse colon were excised, fixed in 4% paraformaldehyde, embedded in paraffin, and sectioned into 5-µm slices. Then, sections were stained with hematoxylin-eosin (H&E) and observed by professional pathologists under optical microscope (Olympus, Tokyo, Japan).

### 2.5. Colonic Immunohistochemical Analysis

Paraffin-embedded tissue sections were stained by immunohistochemistry. The sections were incubated with primary antibody against proliferating cell nuclear antigen (PCNA) at 4 °C overnight, and then with the secondary antibody at room temperature for 30 min. Then, the peroxidase conjugate was visualized with diaminobenzidine solution. Next, the sections were counterstained with hematoxylin for 10–15 s and installed on a coverslip. Finally, the images were obtained by microscope.

### 2.6. Quantitative Real-Time PCR (qRT-PCR)

Total RNA from 10 mg of colonic tissue was homogenized using 800 μL of Trizol reagent, and was reversely transcribed to cDNA using HiScript^TM^Q RT SuperMix. Real-time polymerase chain reaction (PCR) was performed using Hieff^TM^ qPCR SYBR^®^ Green Master Mix on a Bio-rad IQ5 (Bio-Rad Laboratories, Hercules, CA, USA). The level of mRNA was normalized by GAPDH. The 2^−ΔΔ^Ct method was used to process data. Primer sequences were listed in Table 1. 

### 2.7. ELISA

The mouse colons (30 mg) were accurately weighed and reconstituted with PBS. These extracts were then centrifuged for 20 min at 3500 rpm. IL-1β and TNF-α levels were measured in the supernatants using ELISA kits.

### 2.8. Metabolomics Analysis of Serum Samples

#### 2.8.1. Sample Pretreatment

Fifty microliters of serum was mixed with 200 μL of methanol for 5 min, and centrifuged for 25 min. Then, they were placed at −20 °C for 30 min and centrifuged again. Finally, the supernatants were transferred to a new tube for metabolomics research.

#### 2.8.2. UPLC-QTOF-MS/MS Conditions

Chromatographic separation was carried out with a 1290 Infinity system coupled to a 6545 quadrupole time-of-flight (Q-TOF) system (Agilent Technologies, Santa Clara, CA, USA). The separations of samples were performed on a ZORBAX StableBond-C18 column (50 × 2.1 mm, 1.8 μm) at 30 °C. The mobile phase was composed of 0.1% formic acid-water (*v*/*v*; A) and 0.1% formic acid-acetonitrile (*v*/*v*; B) with a 0.3 mL/min flow rate. The following optimal gradient elution condition for serum sample: 0 to 2 min, 5% B; 2 to 5 min, 5 to 40% B; 5 to 10 min, 40 to 55% B; 10 to 15 min, 55% B; 15 to 23 min, 55 to 95% B; 23 to 30 min, 95 to 5% B. The parameters of MS were: drying gas temperature, 350 °C; nebulizing gas (N_2_) flow rate, 11 L/min; sprayer pressure, 35 psi; capillary voltage, 3500 V; scan range, 100–1000 m/z.

#### 2.8.3. Multivariate Data Analysis and Identification of the Differential Metabolites

The chromatographic data were exported and further processed by multivariate statistical analysis with MetaboAnalyst 5.0 (https://www.metaboanalyst.ca/, accessed on 30 September 2022). Then, principal component analysis (PCA) was exploited to obtain an overview of samples. Partial least squares discriminant analysis (PLS-DA) was carried out to discriminate patterns among different groups. Differential metabolites were defined as those with variable importance in projection (VIP) > 1.0 and *p* value less than 0.05. HMDB (http://www.hmdb.ca, accessed on 24 August 2021), MassBank (http://www.massbank.jp, accessed on 24 August 2021) and LIPID MAPS (https://www.lipidmaps.org/, accessed on 24 August 2021) were used for metabolite identification according to the MS and MS/MS data.

### 2.9. Western Blotting

Total proteins in colons of mice were extracted using NP-40 buffer. Then, supernatants were separated by sodium dodecyl sulfate-polyacrylamide gel electrophoresis, and transferred onto nitrocellulose membranes. The membranes were blocked on 5% defatted milk in TBST for 2 h and probed for specific primary antibodies overnight at 4 °C, followed by incubation with the appropriate secondary antibody for 2 h at room temperature. GAPDH antibody was used as the internal reference to ascertain equal loading of proteins. The protein bands were visualized using enhanced chemiluminescent reagent and then analyzed by ImageJ software. 

### 2.10. Cell Culture 

Human colon cancer cells (HT-29, HCT-116 and SW-480) were obtained from the Chinese Academy of Sciences (Shanghai, China). HT-29 cells were cultured in RPMI 1640 medium (Gibco, Carlsbad, CA, USA) supplemented with 10% fetal calf serum, streptomycin (100 U/mL) and penicillin (100 U/mL). HCT-116 cells and SW-480 cells were cultured in DMEM (Gibco, Carlsbad, USA) supplemented with 10% fetal calf serum, streptomycin (100 U/mL) and penicillin (100 U/mL). They were maintained at 37 °C in a humidified environment with 5% CO_2_. In in vitro experiments, SIN and ETX were diluted in complete medium to obtain the required concentrations. 

### 2.11. MTT Assay

The viability of HT-29 cells, HCT-116 cells and SW-480 cells in the presence of SIN was detected by MTT assay. Briefly, HT-29 cells, HCT-116 cells and SW-480 cells (0.8 × 10^4^ cells per well) were seeded in 96-well culture plates, and incubated with SIN (0, 0.625, 1.25, 2.5, 5, 10 and 20 mM) for 24 h. Then, 20 μL of MTT solution (5 mg/mL dissolved in PBS) was added into each well 4 h at 37 °C. The supernatants were then carefully discarded, and 150 μL of dimethyl sulfoxide was added to each well. After shaking for 10 min, the optical absorbance was measured at 570 nm. 

The effect of SIN combined with ETX on the proliferation of HT-29 cells, HCT-116 cells and SW-480 cells was estimated by MTT assay. They were treated with different methods: (1) negative control group (RPMI 1640 culture medium), (2) 2.5 mM SIN group, (3) 50 μM ETX group, (4) 2.5 mM SIN combined with 50 μM ETX group. The other operation steps were the same as above.

### 2.12. Statistical Analysis

The experimental data were presented as mean ± standard error of mean (SEM). One-way analysis of variance (ANOVA) followed by Tukey’s test was performed to compare the differences between multiple groups by using SPSS version 22.0 (SPSS Inc., Chicago, IL, USA). A value of *p* < 0.05 was accepted as a significant difference. 

## 3. Results 

### 3.1. SIN Suppressed Colorectal Tumorigenesis in CAC Mice

**Figure 2:**
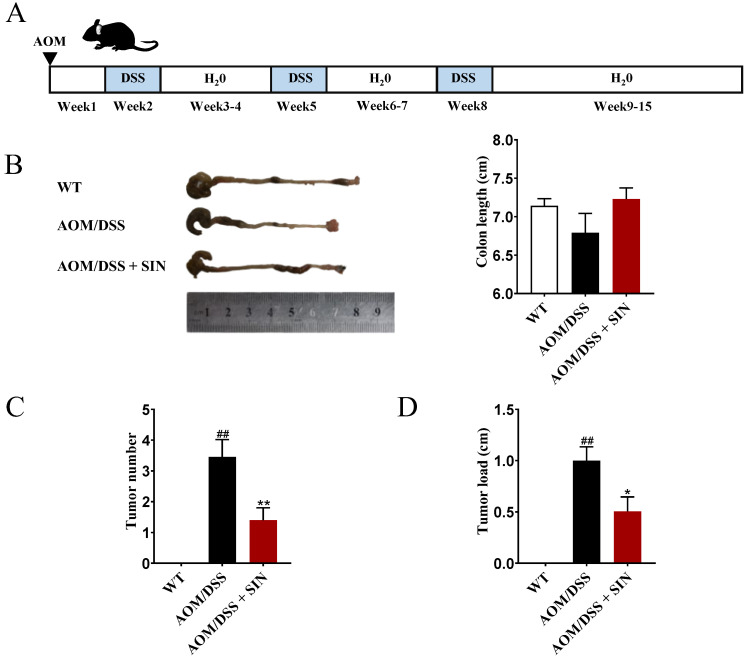
Effect of sinomenine on colitis-associated cancer induced by AOM/DSS in mice. (**A**) Schematic of the AOM/DSS model of colitis-associated cancer. (**B**) The length of the mouse colons. (**C**) The number of tumors in the mouse colon tissues. (**D**) The tumor load in the mouse colon tissues. *n* = 8–11. ^##^*p* < 0.01 vs. WT group. * *p* < 0.05, ** *p* < 0.01 vs. AOM/DSS group.

To investigate the potential function of SIN in CAC, the AOM/DSS-induced CAC mouse model was constructed according to the procedure shown in Figure 2A. The colon length of mice in the AOM/DSS group was shortened, when compared with the wild-type (WT) group (Figure 2B). Such decrease was ameliorated with SIN treatment (Figure 2B). Meanwhile, the incidence of colon adenoma was 100% in the AOM/DSS group and 70% in the AOM/DSS + SIN group. Furthermore, SIN diminished the tumor number and average tumor load by 59.47% and 49.50%, respectively (Figure 2C,D). Together, the mice in the AOM/DSS + SIN group exhibited more rapid recovery, fewer tumors and smaller tumor sizes in the colon than that in the AOM/DSS group.

### 3.2. SIN Attenuated Histopathological Injury in CAC Mice

**Figure 3:**
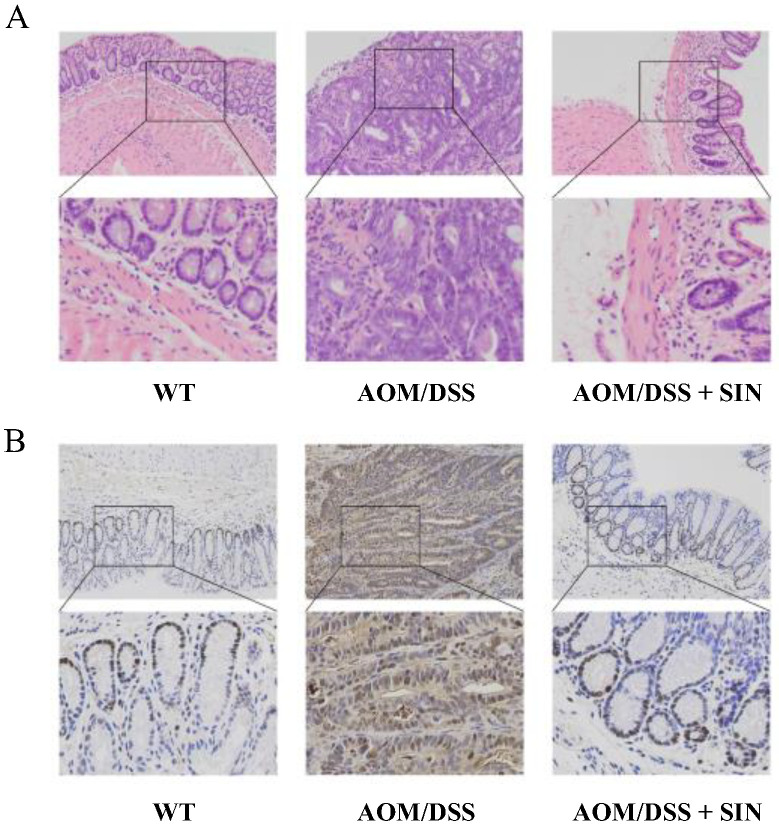
Effect of sinomenine on histopathological injury in CAC mice. (**A**) The histological changes in colonic tissues of mice by H&E staining under a light microscope. (**B**) Infiltration by PCNA^+^ cell was evaluated by immunohistochemistry. The positive cells were dyed in brown. *n* = 8–11.

To gain better insight into the therapeutic effect of SIN, histopathological examination was performed. The results displayed that colonic mucosal damage, necrosis, edema, infiltration of inflammatory cells, nodular hyperplasia of a large number of irregular glands, expanding lumens and canceration of partial hyperplastic glands were observed in the colon of the AOM/DSS group (Figure 3A). SIN markedly alleviated pathological damage, including colonic mucosal injury, necrosis, submucosal edema and infiltration of inflammatory cells, and also dampened the number and size of intramucosal adenocarcinoma (Figure 3A). Functionally, SIN intervention suppressed colitis-associated tumorigenesis in mice. 

### 3.3. SIN Decreased the Number of PCNA^+^ in CAC Mice

PCNA plays a pivotal role in the process of eukaryotic DNA replication, and represents an important index to evaluate the state of cell proliferation. To this end, immunohistochemical assessment was used to evaluate the effect of SIN on colonic PCNA in CAC mice. The results indicated that the number of PCNA^+^ cells induced by AOM/DSS in mice were significantly attenuated by SIN (Figure 3B). Overall, these data further characterized the beneficial role of SIN in CAC.

### 3.4. SIN Alleviated Colonic Inflammation in CAC Mice

**Figure 4:**
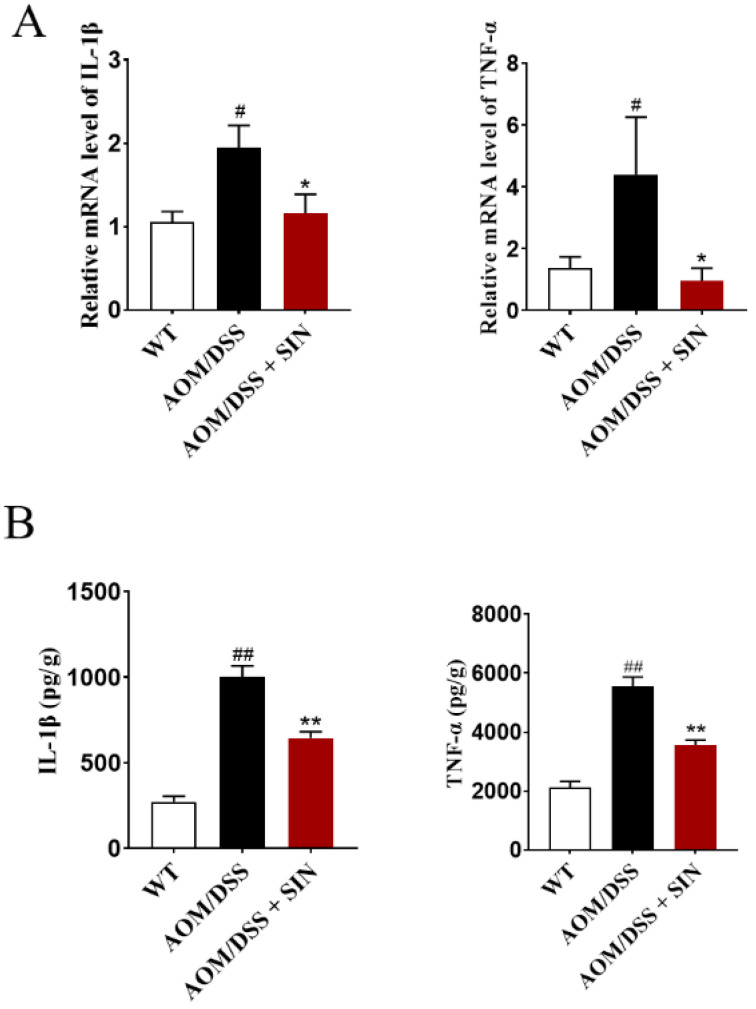
Sinomenine decreased the expression of IL-1β and TNF-α of colon in CAC mice. (**A**) The mRNA levels of IL-1β and TNF-α in colon tissues as determined by qRT-PCR assay. (**B**) The protein levels of IL-1β and TNF-α in colon tissues as determined by ELISA. *n* = 5–11. ^#^ *p* < 0.05, ^##^ *p* < 0.01 vs. WT group. * *p* < 0.05, ** *p* < 0.01 vs. AOM/DSS group.

To probe the anti-CAC effect of SIN in more detail, we interrogated a battery of inflammatory factors, which are hallmark features of CAC. The effects of SIN on the cytokines production in colon of CAC mice were displayed in Figure 4. SIN noticeably downregulated the levels of IL-1β and TNF-α at mRNA and protein levels in colon tissues of CAC mice (Figure 4). These findings collectively elucidated that SIN administration inhibited the production of proinflammatory cytokines in colon of CAC mice.

### 3.5. SIN Modulated Serum Metabolism in CAC Mice

**Figure 5:**
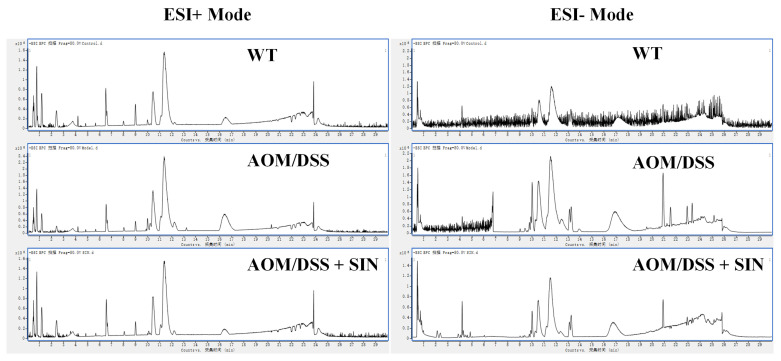
Typical base peak chromatograms of serum samples obtained from the WT, AOM/DSS and AOM/DSS + SIN groups in positive and negative ion mode. Chinese letters in figure image refer to acquisition time.

**Figure 6:**
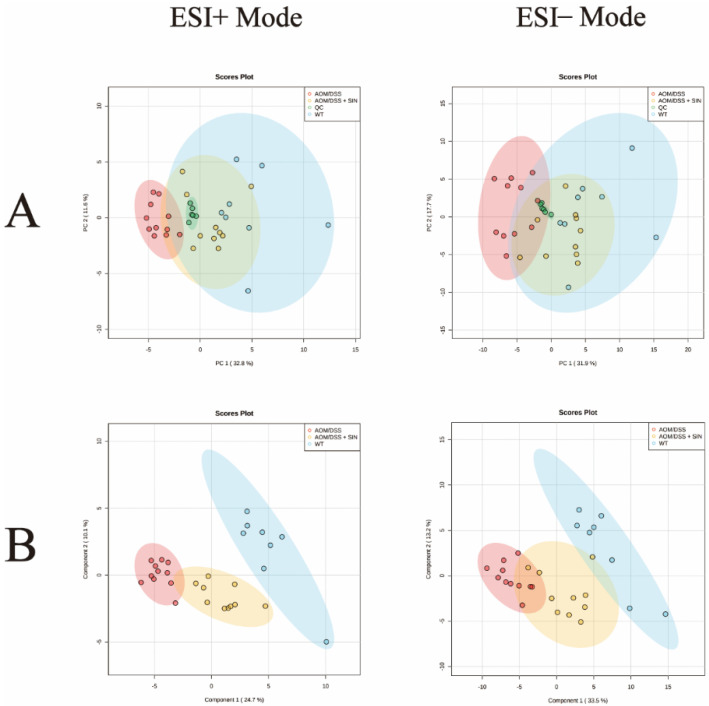
Multivariate statistical analysis of the spectra of serum samples from mice. (**A**) The PCA score plots in positive and negative ion mode. (**B**) The PLS-DA score plots in positive and negative ion mode. *n* = 8–11.

To further dissect the pathogenesis of CAC and decipher the mechanism of SIN against CAC, a metabolomics method based on UPLC-QTOF-MS/MS was employed to identify key endogenous metabolites in serum after SIN intervention in CAC state. Representative base peak chromatograms obtained from serum samples (WT, AOM/DSS and AOM/DSS + SIN group) were depicted in Figure 5. PCA, an unsupervised statistical method, was performed to obtain an overview of the data, eliminate outliers and evaluate metabolomic differences. The PCA score plot showed that the metabolism of the AOM/DSS group was significantly different from that of the other two groups (Figure 6A). In addition, quality control (QC) samples were taken to monitor and control the existing variability [24]. The QC samples were closely clustered together in the PCA score plots of serum samples, indicating that the LC-MS system has good reproducibility and stability (Figure 6A). PLS-DA was employed to describe deep metabolic variations between groups. The results indicated that the AOM/DSS + SIN group was close to the WT group and far away from the AOM/DSS group, suggesting that SIN exerts a metabolic protective effect in CAC (Figure 6B).

**Figure 7:**
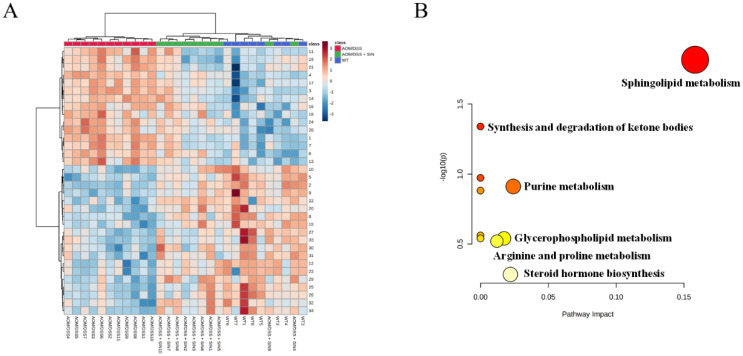
The analysis of metabolic pathway in the treatment of CAC by sinomenine. (**A**) The hierarchical clustering heatmap visualizing the changes in the contents of differential metabolites among the different groups. (**B**) The enrichment analysis of metabolic pathways in the treatment of CAC by sinomenine.

**Figure 8:**
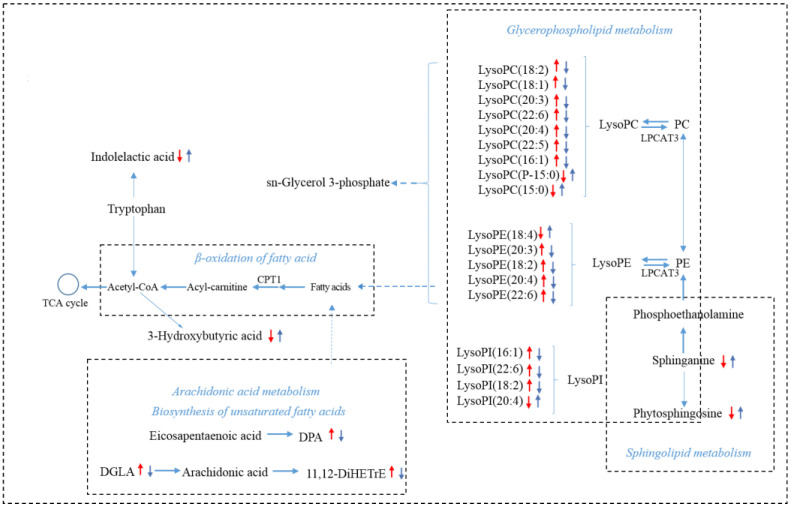
The regulation of sinomenine on lipid metabolism disorders caused by CAC. The alterations in metabolites in the AOM/DSS group compared with the WT group were labeled with a red arrow, and the changes in the AOM/DSS + SIN group compared with the AOM/DSS group were labeled with blue. The upward arrow indicates a significant increase (*p* < 0.05), and the downward arrow indicates a significant decrease (*p* < 0.05).

The variable importance in projection (VIP) and *p* value were combined as the criteria for seeking candidate metabolites. A total of 34 metabolites in serum of CAC mice were perturbed when compared with the WT group (Figure 7A). These endogenous metabolites were reversed back to the normal-like levels after SIN administration (Figure 7A). Table 2 summarized the basic information of these differential metabolites, including name, formula, retention time (RT), detected m/z and trends of different groups. Supplementary Table S1 summarized the detailed information of differential metabolites, including name, RT, formula, database source, electron spray ionization (ESI) mode, mass, MS and MS2 m/z. According to the information of these 34 metabolites, the development of CAC incurred largely metabolic disorders involving fatty acids metabolism, sphingolipid metabolism, purine metabolism and amino acid metabolism (Table 2). SIN intervention could improve multiple metabolic pathways in CAC mice (Figure 7B). To describe the change trend of differential metabolites and relationship of related pathways in CAC mice and SIN-treated mice, a schematic diagram of metabolic pathway was displayed in Figure 8. These results revealed the lipid metabolism of CAC mice was dysregulated and SIN could restore lipid metabolism.

### 3.6. SIN Increased the Expression of CPT1A and LPCAT3 in Colon of CAC Mice

**Figure 9:**
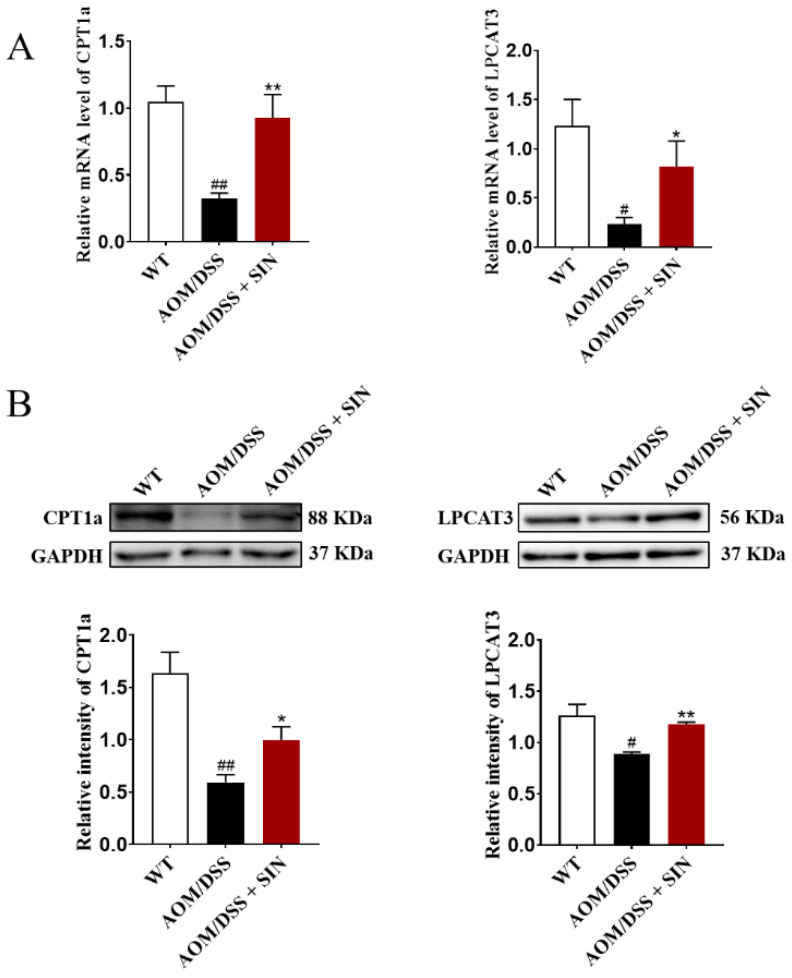
Sinomenine increased the expression of CPT1A and LPCAT3 in the colon of CAC mice. (**A**) The mRNA levels of CPT1A and LPCAT3 in colon tissues as determined by qRT-PCR assay. (**B**) Western blot analysis of the protein expressions of CPT1A and LPCAT3 in colon tissues. *n* = 5–8. ^#^ *p* < 0.05, ^##^ *p* < 0.01 vs. WT group. * *p* < 0.05, ** *p* < 0.01 vs. AOM/DSS group.

SIN consumption reshaped the lipid metabolism in CAC mice revealed by a robustly lower level of lipid identified in the SIN intervention group. Given that CPT1A and LPCAT3 play a central role in lipid metabolic balance, we next queried whether these two enzymes in colon tissue were altered in CAC mice after SIN administration. Both mRNA and protein levels of CPT1A and LPCAT3 in the colon were attenuated in AOM/DSS group relative to their controls, and SIN intervention rescued this decline (Figure 9). Taken together, these results elucidated that SIN augmented expression of CPT1A and LPCAT3, and subsequently accelerated lipid metabolism, thus inhibiting CAC.

### 3.7. Inhibition of CPT1A Weakened the Antiproliferative Effect of SIN

**Figure 10:**
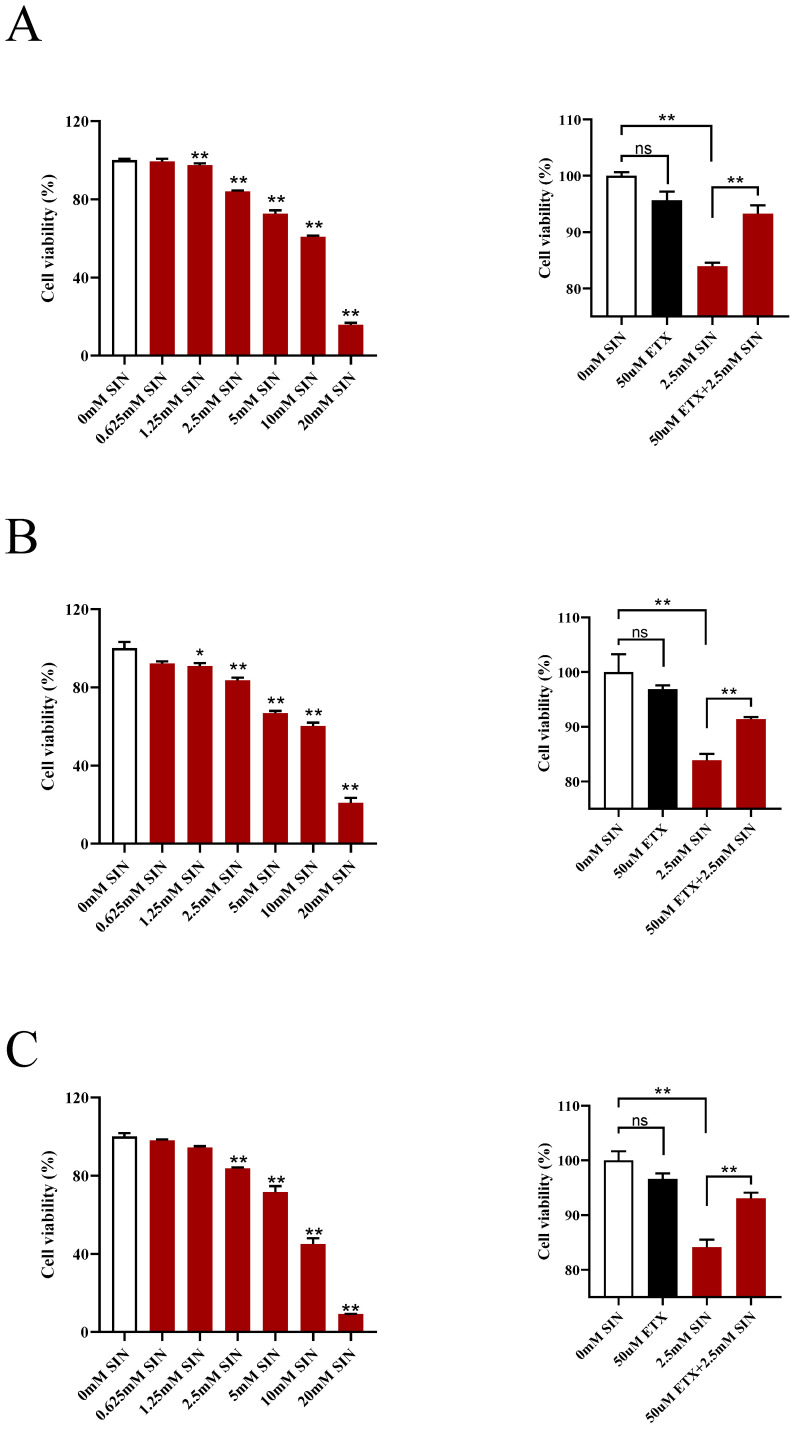
Inhibition of CPT1A weakened the antiproliferative effect of sinomenine. (**A**) MTT assay of proliferation of HT-29 cells incubated with sinomenine and etomoxir. (**B**) MTT assay of proliferation of HCT-116 cells incubated with sinomenine and etomoxir. (**C**) MTT assay of proliferation of SW-480 cells incubated with sinomenine and etomoxir. *n* = 3. * *p* < 0.05, ** *p* < 0.01 vs. the indicated groups.

CPT1A, the rate-controlling enzyme in fatty acid oxidation (FAO), was highly expressed in the SIN intervention group by gene expression and protein expression. Consequently, we wanted to find out whether inhibition of CPT1A would attenuate the antiproliferative effect of SIN. Therefore, a pharmacological approach to inhibit lipid metabolism by dampening CPT1A activity via ETX was adopted. The results deciphered that the proliferation of human colon cancer cells (HT-29, HCT-116 and SW-480) treated with SIN decreased in a dose-dependent manner after incubation for 24 h (Figure 10). Although ETX had no effect on the proliferation of colon cancer cells, the proliferation of colon cancer cells incubated with ETX and SIN was increased robustly compared with SIN alone, supporting the notion that pharmacologic disruption of CPT1A blunted the antiproliferative effect of SIN. As a result, the beneficial effects of SIN could be transmitted through FAO, and the antiproliferative effect of SIN on CAC may be dependent on CPT1A.

## 4. Discussion

CAC poses a severe challenge to health-care systems worldwide; thereby novel and effective drugs need to be developed. Despite multiple lines of evidence illustrating that SIN inhibited the growth and metastasis of human colon carcinoma cells, its potential role and mechanism in CAC are still ambiguous [16,17]. The AOM/DSS-induced mouse model, which was sufficient to emulate the clinicopathological characteristics of human CAC, is usually used to investigate the efficacy of drugs. Based on this notion, the therapeutic effect of SIN on the CAC animal model was evaluated. The significant differences in colon length, incidence of colonic adenoma, the tumor number, average tumor load, and histological changes of colon between the WT group and the AOM/DSS group confirmed that the establishment of the CAC model was successful. According to the results of pharmacodynamic experiments, SIN markedly ameliorated the symptoms of CAC mentioned above.

The established and powerful connection between inflammatory cytokines and CAC has been widely recognized. Inflammatory cytokines may facilitate inflammation formation, tumor growth and metastasis through multiple molecular mechanisms, which supported the notion that inflammatory cytokines are crucial for the maintenance of the tumor microenvironment. It has been confirmed that the level of proinflammatory factors in tumor tissue of patients with CAC was increased. In addition, TNF-α-deficient mice with CAC showed a reduction in mucosal damage after AOM/DSS treatment, followed by alleviation of colonic tumors [25]. The increased secretion of IL-1β will lead to the production and maintenance of an inflammatory microenvironment surrounding cancer cells, which is conducive to tumor [26]. Thus, attenuation of inflammatory cytokine production seems to be an effective method to prevent and treat CAC. To address this scenario, we interrogated a battery of inflammatory factors. The colons of SIN-administered AOM/DSS-treated mice displayed noticeably lower TNF-α and IL-1β expression compared with AOM/DSS-treated mice. These findings demonstrated that SIN could mitigate colonic inflammation in CAC mice, thereby inhibiting tumor growth.

Although our initial study implicated that SIN attenuated CAC functionally, the mechanism in vivo remains unsettled. Untargeted metabolomics results revealed the potential targets of SIN and mechanisms responsible for its chemopreventive activity. In this study, 34 differential metabolites related to CAC were observed. Specifically, the abundance of lysoPI (16:1), lysoPC (18:2), lysoPC (18:1), dihomo-gamma-linolenic acid, lysoPC (22:6), lysoPC (20:4), lysoPE (20:3), lysoPE (18:2), docosapentaenoic acid, lysoPC (22:5), lysoPC (20:3), lysoPE (20:4), lysoPE (22:6), lysoPI (22:6) and lysoPI (18:2) were markedly elevated, while the abundance of phytosphingosine, lysoPE (18:4), 21-deoxycortisol, sphinganine, creatine, inosine, 2-aminocaprylic acid, phenylacetylglycine, lysoPI (20:4), hypoxanthine, acetyl-DL-leucine, acetylphenylalanine, leucinic acid, p-cresol sulfate, indolelactic acid, 3-hydroxybutyric acid, allantoin, indoxyl sulfate and 2-hydroxyvalerate were notably reduced in CAC mice. However, these endogenous metabolites returned to normal levels after SIN intervention. Notably, lysoPC and lysoPE accounted for 65% of all changed metabolites, so it is speculated that the therapeutic effect of SIN could be attributed to the normalization of lipid metabolism. 

Mitochondrial FAO is the main pathway of fatty acid catabolism and plays an important role in maintaining whole-body energy balance. CPT1 is a rate limiting enzyme responsible for the conveyance of lysoPC and lysoPE for β-oxidation. CPT1A is the most widely distributed and active member in CPT1 family. LPCAT catalyzes acylation of lysoPC to produce phosphatidylcholine. LPCAT3 is the most abundant isoform of LPCAT in the intestine. Once LPCAT3 is inhibited, which may lead to the excessive lysoPC accumulation, thus aggravating inflammation. Similarly, disruption of LPCAT3-dependent phospholipid noticeably augments tumor formation in Apc^min^ mice [27]. Therefore, the effect of SIN on the expression of CPT1A and LPCAT3 was assessed to decipher the biochemical mechanism of SIN intervention in CAC. Intriguingly, the expression of CPT1A and LPCAT3 in colon was found to be dramatically enhanced by SIN intervention, lending support to the hypothesis that SIN promoted the expansion of CPT1A and LPCAT3 in mouse colon tissue. Indeed, overexpression of CPT1A in numerous cancers was observed; rather, the expression level of CPT1A was at significantly low level in patients with CRC [28]. Herein, we report that both mRNA and protein levels of CPT1A in the colon were attenuated in model groups relative to their controls, and SIN intervention rescued this decline.

Emerging studies have suggested the enhancement of FAO inhibits the proliferation of colon cancer cells, and CPT1A is a key enzyme responsible for FAO, raising speculation that SIN inhibited tumor cell proliferation via CPT1A [29]. To verify this hypothesis, human colorectal cancer cells were cultured in vitro and subsequently treated with SIN and ETX. The results of in vitro experiments confirmed that ETX counteracted the inhibitory effects of SIN in human colorectal cancer cells. In short, SIN suppressed CAC progression via CPT1A-mediated FAO.

Herein, we elucidated the effect of SIN on serum metabolism from the perspective of intestine. On the one hand, colon tissue is the site of colon cancer. On the other hand, accumulating evidence suggests that the action of SIN is gut-dependent [30,31,32,33]. Consequently, colonic tissues were selected to investigate the changes of metabolic enzymes of differential metabolites in this study. Interestingly, 3-hydroxybutyric acid, as one of the endogenous metabolites interfered by SIN, is produced in the liver rather than in the intestine, raising speculation that SIN may ameliorate CAC by regulating systemic metabolism. Future investigation is warranted to clarify whether the anti-CAC effect of SIN depends on intestinal regulation or systemic regulation.

## 5. Conclusions

In the present study, a comprehensive strategy of pharmacodynamics, metabolomics and molecular biology was utilized to clarify the efficacy and potential mechanism of SIN in the treatment of CAC. Our work interpreted that SIN attenuated CAC through inhibiting tumor formation, reducing inflammation, restoring histological injury and correcting abnormal lipid metabolism, laying a foundation for the application of SIN in the treatment of CAC. Importantly, 34 endogenous metabolites of CAC mice were reversed back to the normal-like levels under the action of SIN. According to these differentially expressed metabolites, SIN mainly achieved a certain effect on regulating the disorder of lipid metabolism. Notably, we demonstrated mechanistically that the protective mechanism of SIN might be attributed to the FAO mediated by CPT1A, thereby alleviating CAC. Collectively, our study cast a spotlight on the pathogenesis of CAC from the metabolic perspective, and SIN holds the capacity to be developed as a new candidate drug against CAC in the clinic.

## Figures and Tables

**Table 1:** Mouse primer pairs used in real-time polymerase chain reaction.

Gene	Primer	Sequences
*IL-1β*	Forward	5′- TCGCAGCAGCACATCAACAAGAG -3′
	Reverse	5′- AGGTCCACGGGAAAGACACAGG -3′
*TNF-α*	Forward	5′- GCCTCTTCTCATTCCTGCTTGTGG -3′
	Reverse	5′- GTGGTTTGTGAGTGTGAGGGTCTG -3′
*CPT1A*	Forward	5′- GATGTGGACCTGCATTCCTT -3′
	Reverse	5′- TCCTTGTAATGTGCGAGCTG -3′
*LPCAT3*	Forward	5′- GCTGCGGCTCATCTTCTCCATC -3′
	Reverse	5′- AGGAACTGAAGCACGACACATAGC -3′
*GAPDH*	Forward	5′- GGTTGTCTCCTGCGACTTCA -3′
	Reverse	5′- TGGTCCAGGGTTTCTTACTCC -3′

**Table 2:** The potential differential metabolites identified in serum which can be regulated by sinomenine (Δppm < 20).

No.	RT (min)	Formula	Identification	Detected m/z	Change Trend (AOM/DSS vs. WT)	Change Trend (AOM/DSS + SIN vs. AOM/DSS)
1	10.42	C_25_H_47_O_12_P	LysoPI (16:1)	571.2961	↑ ***	↓ ***
2	6.62	C_18_H_39_NO_3_	Phytosphingosine	318.2997	↓ ***	↑ ***
3	10.2	C_26_H_50_NO_7_P	LysoPC (18:2)	520.3393	↑ ***	↓ **
4	12.23	C_26_H_52_NO_7_P	LysoPC (18:1)	522.3552	↑ ***	↓ ***
5	8.99	C_23_H_40_NO_7_P	LysoPE (18:4)	474.2596	↓ ***	↑ **
6	21.52	C_20_H_34_O_2_	Dihomo-gamma-linolenic acid	307.262	↑ ***	↓ *
7	10.23	C_30_H_50_NO_7_P	LysoPC (22:6)	568.3392	↑ ***	↓ **
8	6.43	C_21_H_30_O_4_	21-Deoxycortisol	347.2217	↓ ***	↑ **
9	8	C_18_H_39_NO_2_	Sphinganine	302.3051	↓ ***	↑ ***
10	0.49	C_4_H_9_N_3_O_2_	Creatine	132.0766	↓ ***	↑ **
11	10.23	C_28_H_50_NO_7_P	LysoPC (20:4)	544.339	↑ ***	↓ **
12	0.82	C_10_H_12_N_4_O_5_	Inosine	269.089	↓ ***	↑ **
13	10.75	C_25_H_46_NO_7_P	LysoPE (20:3)	504.3049	↑ ***	↓ **
14	9.87	C_23_H_44_NO_7_P	LysoPE (18:2)	478.2923	↑ ***	↓ **
15	0.51	C_8_H_17_NO_2_	2-Aminocaprylic acid	160.1333	↓ ***	↑ **
16	21.01	C_22_H_34_O_2_	Docosapentaenoic acid	331.263	↑ ***	↓ *
17	11.11	C_30_H_52_NO_7_P	LysoPC (22:5)	570.3522	↑ ***	↓ **
18	11.38	C_28_H_52_NO_7_P	LysoPC (20:3)	546.3551	↑ **	↓ *
19	9.96	C_25_H_44_NO_7_P	LysoPE (20:4)	502.2921	↑ **	↓ **
20	4.23	C_10_H_11_NO_3_	Phenylacetylglycine	194.0805	↓ **	↑ **
21	9.98	C_27_H_44_NO_7_P	LysoPE (22:6)	526.2919	↑ **	↓ **
22	20.52	C_29_H_49_O_12_P	LysoPI (20:4)	621.3089	↓ *	↑ ***
23	0.82	C_5_H_4_N_4_O	Hypoxanthine	137.0467	↓ *	↑ **
24	10.08	C_31_H_49_O_12_P	LysoPI (22:6)	643.2888	↑ ***	↓ *
25	4.6	C_8_H_15_NO_3_	Acetyl-DL-Leucine	172.0972	↓ ***	↑ *
26	4.65	C_11_H_13_NO_3_	Acetylphenylalanine	206.0823	↓ ***	↑ *
27	4.065	C_6_H_12_O_3_	Leucinic acid	131.0715	↓ ***	↑ *
28	9.95	C_27_H_49_O_12_P	LysoPI (18:2)	595.2885	↑ **	↓ *
29	4.72	C_7_H_8_O_4_S	p-Cresol sulfate	187.0071	↓ **	↑ **
30	4.78	C_11_H_11_NO_3_	Indolelactic acid	204.0668	↓ **	↑ *
31	0.91	C_4_H_8_O_3_	3-Hydroxybutyric acid	103.0403	↓ **	↑ **
32	0.52	C_4_H_6_N_4_O_3_	Allantoin	157.0369	↓ **	↑ **
33	4.14	C_8_H_7_NO_4_S	Indoxyl sulfate	212.0011	↓ *	↑ *
34	2.1152	C_5_H_10_O_3_	2-Hydroxyvalerate	117.0561	↓ *	↑ *

The change trend of differentially expressed metabolites was labeled with (↑) up-regulation and (↓) down-regulation (*, *p* < 0.05; **, *p* < 0.01; ***, *p* < 0.001).

## Data Availability

The datasets analyzed in the current study are available from the corresponding author upon request.

## Supplementary Materials

The following supporting information can be downloaded at: https://www.mdpi.com/article/10.3390/metabo12100946/s1, Table S1: Summary of significantly differential metabolites identified in serum which can be regulated by sinomenine based on UPLC-QTOF-MS/MS.

## Abbreviations

AOM: azoxymethane; CAC, colitis-associated cancer; CPT1A, carnitine palmitoyltransferase 1A; CRC, colorectal cancer; DSS, dextran sulfate sodium; ELISA, enzyme-linked immunosorbent assay; ESI, electron spray ionization; ETX, etomoxir; FAO, fatty acid oxidation; H&E, hematoxylin and eosin; LPCAT3, lysophosphatidylcholine acyltransferase 3; MTT, 3-(4,5-dimethylthiazol-2-yl)-2,5-diphenyl tetrazolium bromide; PCA, principal component analysis; PCNA, proliferating cell nuclear antigen; PLS-DA, partial least squares discriminant analysis; SIN, sinomenine; UPLC-QTOF-MS/MS, ultra-performance liquid chromatography coupled with quadrupole time-of-flight mass spectrometry; VIP, variable importance in projection.
